# Cortical blindness as severe neuro-ophthalmological manifestation of tuberous sclerosis complex

**DOI:** 10.3205/oc000062

**Published:** 2017-04-19

**Authors:** Alvaro I. Ortiz Z., Pedro Luis Cárdenas, Luis C. Escaf, Marcela Peralta

**Affiliations:** 1 Neuro-Ophthalmology Department, Fundación Oftalmológica de Santander – FOSCAL, Bucaramanga, Colombia; 2 Aljaorza S.A. Ophthalmology Center, Machala, Ecuador; 3 Fundación Oftalmológica de Santander – FOSCAL, Bucaramanga, Colombia; 4 Critical Care Department, Fundación Oftalmológica de Santander – FOSCAL, Bucaramanga, Colombia

**Keywords:** tuberous sclerosis, blindness, hamartoma, retina, electrophysiology

## Abstract

Patients with retinal lesions related to tuberous sclerous complex (TSC) commonly have no impairment of visual acuity. We present a case of a 1-year-old Hispanic girl with TSC in which bilateral cortical blindness is documented.

## Introduction

Initially described by von Recklinghausen in 1862, tuberous sclerous complex (TSC) is a genetic, multisystem, neurocutaneous disorder that affects any race or gender, characterized by the potential formation of benign, non-invasive lesions “hamartomas” in various organs without increased risk for malignancies [1], [2], [3]. It is classified within neurocutaneous disorders along with neurofibromatosis type 1 and 2, schwannomatosis, von-Hippel-Lindau syndrome, and others. Recognizing the presence of one of this disorders is important for clinical care, treatment and genetic counseling [4]. The term “tuberous sclerosis complex” was coined by Moolten in 1942 because of the systemic manifestations of the disease [5]. TSC is an autosomal dominant disorder with variable expression and incomplete penetrance, though two-thirds of patients have sporadic or de novo mutations. The prevalence is 1/5.800 to 1/15.000, therefore TSC is one of the most common genetic diseases [6], [7], [8], [9].

TSC is characterized by the presence of multiple hamartomas, most notably in the skin, brain, kidneys, heart, and eyes. After dermatological findings, neurological manifestations are most frequently seen. The spectrum ranges from patients with normal intellect and no seizures to severe mental retardation, incapacitating seizures and behavior disorders [1], [2], [4], [5]. The severity of the neurological and renal manifestations is the most important predictor for quality of life, morbidity, and mortality in patients with TSC. Ophthalmic manifestations were found in up to 50% of cases and can affect any part of the eye, retinal hamartomas are the most common retinal manifestation. Neuro-ophthalmologic disorders are rare and usually mild. Visual acuity is respected in most cases, although visual field defects have been reported depending on the location of the tubers [10]. In our case, we show the rare, early, and diffuse impairment of the visual pathway and the central nervous system with a cortical blindness.

## Case description

**Figure 1:**
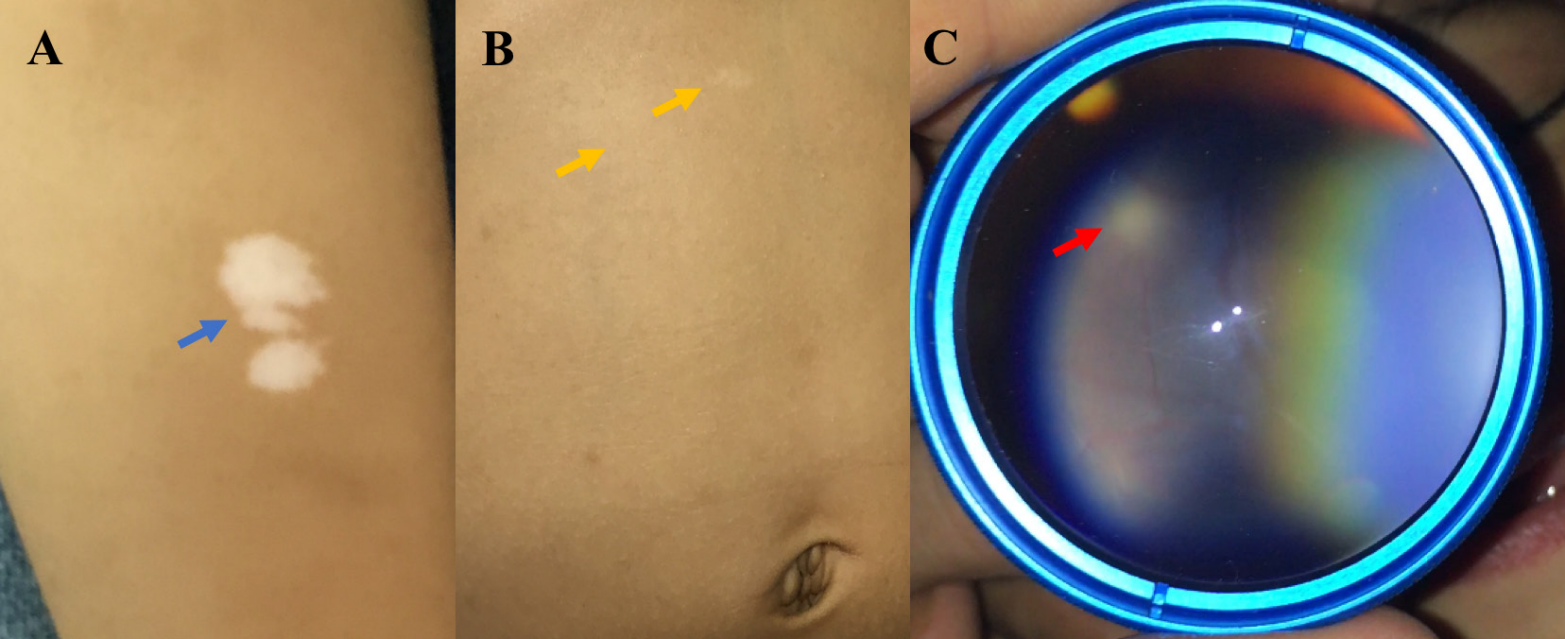
A) Hypopigmented macules on lower leg (blue arrow). B) Confetti skin lesions (yellow arrow). C) Retinal Hamartoma superior to disc in her rigth eye (red arrow).

**Figure 2:**
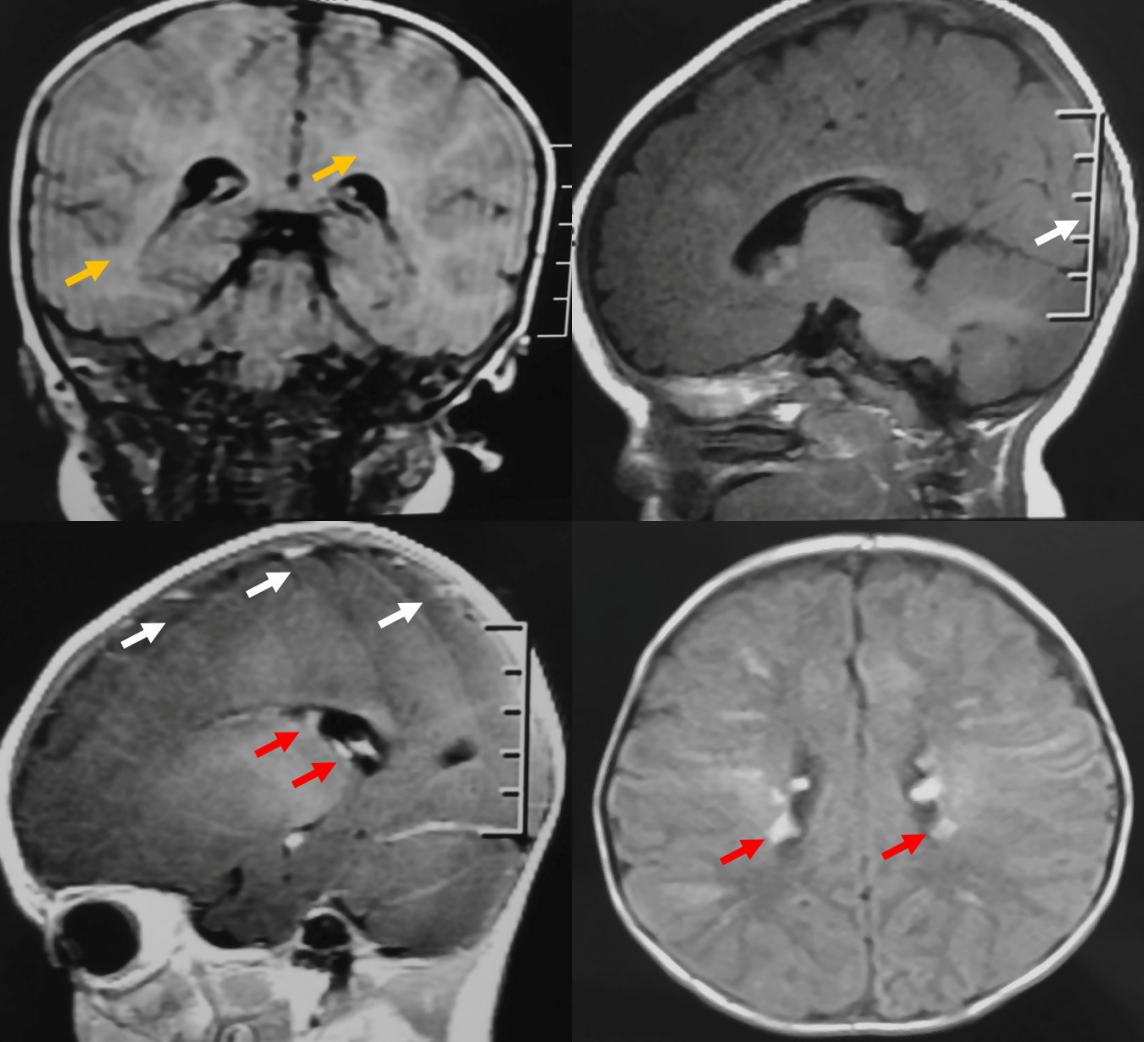
Cerebral MRI revealing diffuse areas of commitment in the white matter (yellow arrow) and cortex with multiple frontal, temporal, parietal, and occipital (white arrow), subependymal lesions of tubers (red arrow).

**Figure 3:**
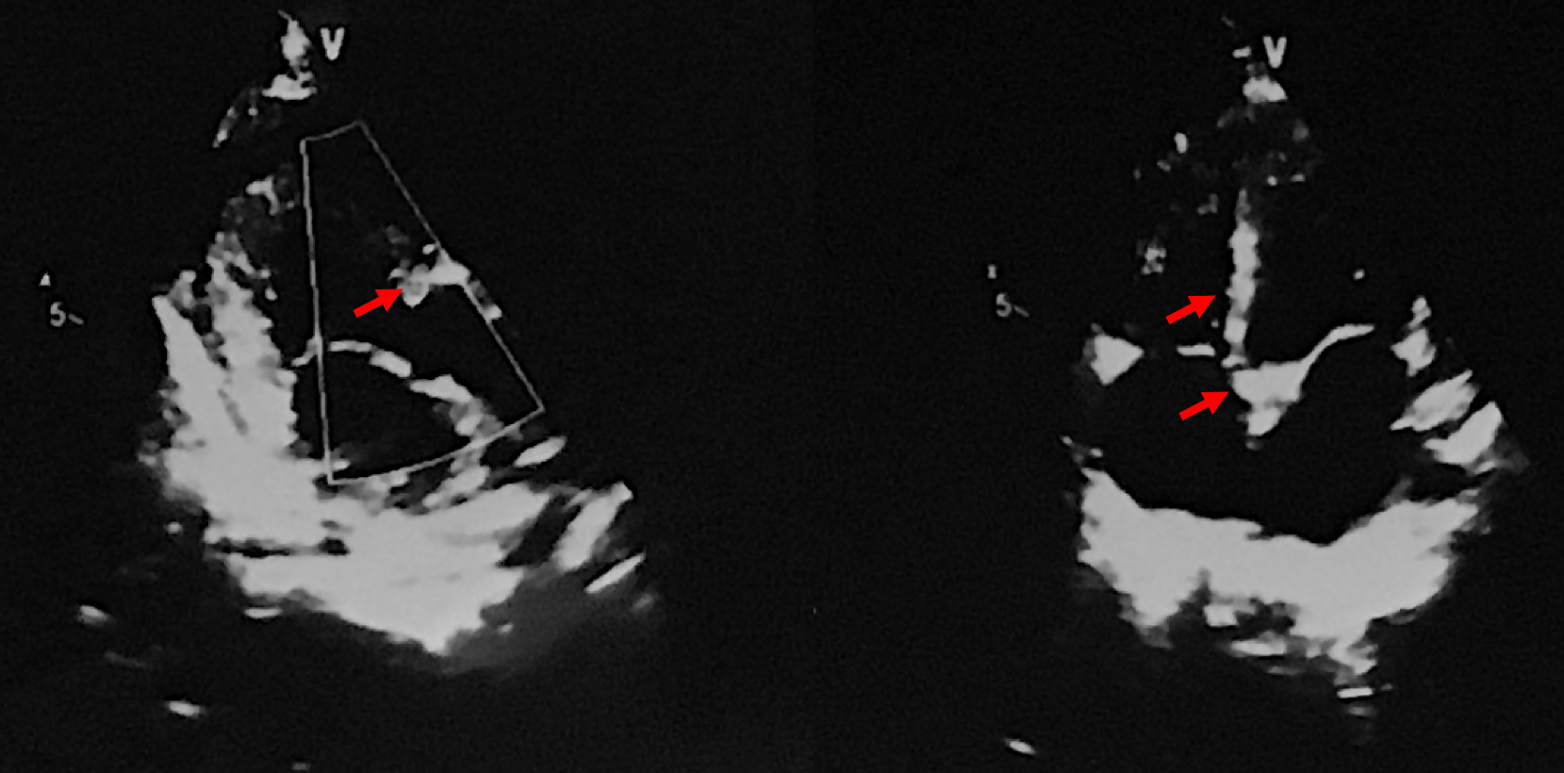
Echocardiogram revealed the rhabdomyoma that compromised interventricular septum, left ventricle and aortic valves (red arrow).

A 1-year-old girl was brought to our neuro-ophthalmology department because of poor fixation of objects during the last 7 months (no pregnancy or family history was significant). She had a history of uncontrolled seizures and cardiological symptoms being diagnosed with rhabdomyoma and West syndrome. On dermatologic inspection, she had multiple hypopigmented macules on her lower legs (Figure 1A (Fig. 1)) and “confetti” skin lesions on the abdomen (Figure 1B (Fig. 1)). On examination, there was bilateral poor fixation and no interaction with the environment in presence of visual stimuli, the pupillary reflexes were normal. Slit-lamp examination was unremarkable. On fundus examination, the right eye had a lesion of 1 disk diameter, yellowish and elevated, superior to the disk compatible with a retinal hamartoma (Figure 1C (Fig. 1)) and another in the peripheral retina; in the left eye, another semi translucent lesion was seen superior to the vascular arcade. Brain contrast enhanced MRI revealed diffuse areas of commitment in the white matter and cortex with multiple subependymal lesions suggestive of tubers (Figure 2 (Fig. 2)). The echocardiogram showed a rhabdomyoma that compromised the interventricular septum essentially (Figure 3 (Fig. 3)). The flash visual evoked potentials (VEP) revealed bilateral marked increased latency with respected amplitude in both eyes. With all these manifestations and applying the clinical criteria of the international tuberous sclerosis complex consensus of 2012, we confirmed the diagnosis of tuberous sclerosis complex (TSC) and cortical blindness. 

**Figure 4:**
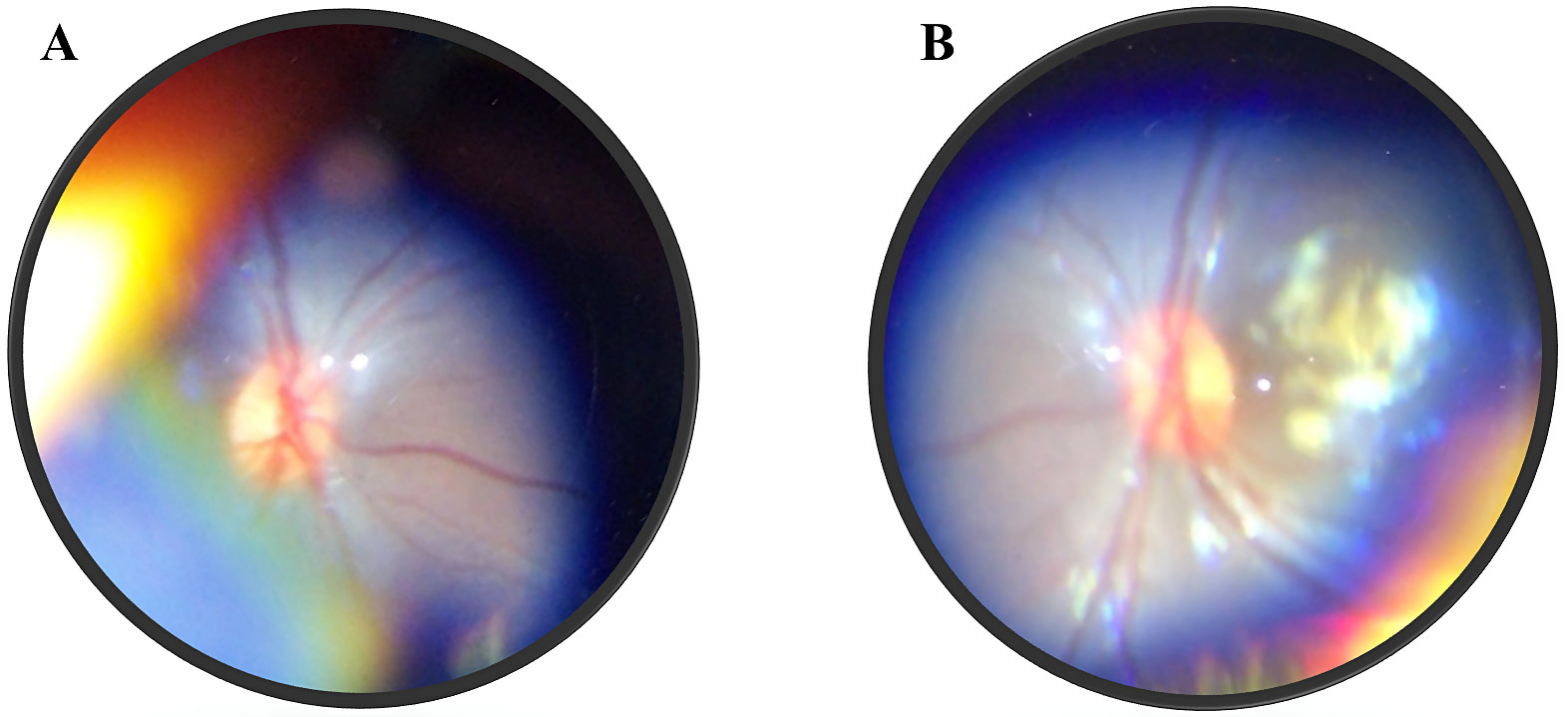
Color photographs of the optic nerve at 1-year follow-up. Note the normal appearance of the optic disc and neuroretinal rim.

At 1-year follow-up visit, the mother reported persistence of seizures despite medical treatment. The girl had absence of fixation-follow-maintain monocular/binocular pattern and inadequate interaction with environment in presence of visual stimuli. The presence of oculodigital reflex in both eyes was also documented. The anterior segment and intraocular pressure were within normal limits. A concomitant pendular nystagmus was evident in both eyes. Fundus examination revealed healthy optical discs with a normal neuroretinal rim (Figure 4 (Fig. 4)), the previously described lesions presented no changes. New electrophysiological studies were carried out, flash VEP showed a deepening of the defect given by a marked prolongation of the latency.

## Discussion

TSC is a genetic, multisystem, neurocutaneous disorder characterized by the potential formation of hamartomas in various organs and systems including the central nervous system (CNS) and visual pathways [9]. The two most important gene mutations described are abnormalities found in TSC1 and TSC2 which code for proteins hamartin and tuberin. TSC features a large variety of clinical manifestations and severity among patients even with identical genotypes. With the appearance of new and cheaper genetic diagnostic techniques, overall mutation detection rate in patients with TSC is as high as 85–90% [9], [10].

According to the International Consensus Conference 2012, definite or possible TSC diagnosis can be made. For the definite diagnosis of TSC, two major features or one major feature and ≥2 minor features have to be present. For a possible diagnosis, 1 major feature or ≥2 minor features should be found [2], [10]. Our patient complied four major clinical features cortical dysplasia, subependymal nodules, cardiac rhabdomyoma, and multiple retinal hamartomas as well as two minor clinical features “confetti” skin lesions and retinal achromic patch, given a definite diagnosis of TSC. 

The clinical presentation includes hamartomas that usually compromise a great variety of systems, being CNS the most common, leading to seizures and mental retardation, depending on the localization of the tubers in the cortex and related closely with convulsive episodes [8], [9]. Other findings like cutaneous and renal manifestations are not uncommon, others like cardiac manifestations were seen in 1% of the cases [8]. One report of 100 patients with TSC between 2 to 76 years [9] showed that ophthalmic manifestations can be divided into retinal and non-retinal. Retinal features are the most common manifestation, identified in approximately 44% of individuals. Retinal hamatormas are known to have diverse morphological presentation ranging from retinal astrocytic hamartomas like a translucent lesion to multilobular mulberry lesion and transitional lesions that in some cases can compromise the optic disk and in others cases it may bleed and cause a vitreous hemorrhage. Non-retinal manifestations like angiofibromas of eyelids, iris, and lens coloboma have been described [9], [10]. The neuro-ophthalmological manifestations are described as variable degrees of papilledema in the setting of optic disk hamartoma, paralytic and non-paralytic strabismus, and focal impairment of the visual pathway manifesting itself as visual field defects, which in some patients may manifest as decreased visual acuity [8], [10]. These lesions, especially retinal, are not uncommon to be associated with partial or small impairment of visual acuity according to their location and insignificant progression over time [10]. We hereby report a case of bilateral blindness as the presenting feature of TSC for early, rare, and diffuse impairment of the visual pathway in the occipital cortex. 

## Conclusions

This case report showed an early, diffuse, and marked central nervous system and visual pathway impairment leading to cortical blindness in our patient. The follow-up is important to identify the early onset or growth of tumors of different location. 

## Notes

### Competing interests

The authors declare that they have no competing interests.
